# Reprogramming the Immunosuppressive Microenvironment in Glioblastoma Through Oncolytic Herpes Simplex Virus Therapy: A Systematic Review

**DOI:** 10.3390/cells15100867

**Published:** 2026-05-09

**Authors:** Kamil Poboży, Zuzanna Ząbek, Grzegorz Turek, Jakub Litak, Binbin Zhu, Patrycja Gierszon, Joanna Litak, Michał Szymoniuk, Justyna Zielińska-Turek, Grzegorz Staśkiewicz, Kamil Torres, Mirosław Ząbek, Wojciech Czyżewski

**Affiliations:** 1 Department of Neurosurgery, Brodnowski Masovian Hospital, 03-242 Warsaw, Poland; pobozykamil@gmail.com (K.P.);; 2 Clinical Immunology Student Scientific Association, Medical University of Warsaw, 02-006 Warsaw, Poland; 3 Department of Clinical Immunology, Medical University of Lublin, 20-093 Lublin, Poland; jakub.litak@gmail.com; 4 Anesthesiology Department, The First Affiliated Hospital of Ningbo University, Ningbo 315020, China; pingchi1983@126.com; 5 Faculty of Medical Sciences, University of Social and Medical Sciences in Lublin, 20-102 Lublin, Poland; 6 St. John’s Cancer Center, 20-090 Lublin, Poland; 7 Department of Neurosurgery, T. Marciniak Lower Silesian Specialist Hospital, 54-049 Wroclaw, Poland; michmatsz@gmail.com; 8 Department of Neurology, National Medical Institute of the Ministry of Interior and Administration, 02-507 Warsaw, Poland; jzturek@gmail.com; 9 1st Department of Radiology, Medical University of Lublin, 20-090 Lublin, Poland; 10 Department of Plastic, Reconstructive Surgery with Microsurgery, Medical University of Lublin, 20-090 Lublin, Poland; 11 Gamma Knife Center, 03-242 Warsaw, Poland; 12 Department of Neurosurgery, Centre of Postgraduate Medical Education, 03-242 Warsaw, Poland; 13 Department of Neurosurgery, Maria Sklodowska-Curie National Research Institute of Oncology, 02-781 Warsaw, Poland; wojciech.w.czyzewski@gmail.com; 14 Department of Didactics and Medical Simulation, Medical University of Lublin, 20-093 Lublin, Poland

**Keywords:** glioblastoma, oncolytic herpes simplex virus, tumor microenvironment, immunosuppression, immunomodulation, oncolytic virotherapy, cancer immunotherapy, neurooncology

## Abstract

**Highlights:**

**What are the main findings?**

- Current evidence suggests that oncolytic herpes simplex virus therapy may reshape the glioblastoma tumor microenvironment through immune, vascular, and stromal remodeling in addition to direct tumor cell lysis.
- Durable therapeutic responses in experimental models appear to depend on coordinated activation of adaptive immunity and interception of virus-induced resistance pathways, particularly within myeloid and endothelial compartments.

**What are the implications of the main findings?**

- oHSV may be better understood as a multifunctional immunomodulatory platform rather than solely as a single-agent cytolytic therapy.
- Future clinical development should emphasize rational combination strategies and microenvironment-aware treatment design, while acknowledging that clinical validation remains limited.

**Abstract:**

Glioblastoma (GBM) is characterized by a profoundly immunosuppressive tumor microenvironment that limits the efficacy of conventional and immune-based therapies. Oncolytic herpes simplex virus (oHSV) therapy has emerged as a strategy capable of both tumor-selective infection and immune microenvironment modulation. This systematic review aimed to synthesize current evidence on how oHSV therapy reshapes the immunosuppressive microenvironment of GBM. A systematic search of PubMed (MEDLINE) and Embase identified original studies published between 2016 and 2025 investigating immunological or microenvironmental effects of oHSV-based therapies in GBM (final search: 28 January 2026). Preclinical and clinical studies were included, whereas reviews, editorials, conference abstracts, and studies of non-herpes oncolytic viruses were excluded. Study selection and data extraction were performed independently by two reviewers. Due to heterogeneity in models, viral constructs, and outcome measures, a qualitative narrative synthesis was conducted. Of 214 records, 22 studies met the inclusion criteria. Most were preclinical studies using orthotopic immunocompetent murine models, with limited clinical evidence including an early-phase trial in recurrent high-grade glioma. Therapeutic efficacy frequently correlated with tumor microenvironment remodeling rather than viral replication alone. oHSV infection promoted inflammatory signaling, antigen presentation, macrophage polarization, and effector T-cell recruitment but also induced counter-regulatory mechanisms such as myeloid-mediated immunosuppression and vascular or stromal barriers. The clinical significance and durability of these effects remain to be established in larger prospective studies. Despite heterogeneous designs and limited clinical data, current evidence suggests that oHSV may function as a platform for immune microenvironment reprogramming in glioblastoma.

## 1. Introduction

Glioblastoma (GBM) is a primary malignancy of the central nervous system with median survival rarely exceeding 15 months despite maximal surgical resection followed by radiotherapy and temozolomide-based chemotherapy [1,2]. Over the past decade, extensive molecular characterization has transformed the understanding of GBM, yet this knowledge has translated into only marginal clinical progress [1,2,3,4]. One of the central barriers to effective therapy is the profoundly immunosuppressive tumor microenvironment (TME), which actively excludes or disables antitumor immune responses and renders glioblastoma largely refractory to systemic immunotherapies that have revolutionized treatment of other solid tumors [5,6,7].

Unlike immunologically “hot” cancers, GBM is characterized by low T-cell infiltration, extensive myeloid dominance, dysfunctional antigen presentation, and strong local immunoregulatory signaling [8,9]. Tumor-associated macrophages and microglia constitute the majority of immune cells within the glioblastoma niche and promote tumor growth through immunosuppression, angiogenesis, and extracellular matrix remodeling [10,11]. In parallel, the blood–brain barrier, abnormal vasculature, and extensive stromal crosstalk further restrict immune cell trafficking and drug penetration [12,13]. These features collectively explain the limited clinical efficacy of immune checkpoint inhibitors, cancer vaccines, and adoptive cell therapies in unselected GBM populations and emphasize the importance of approaches capable of reshaping the local ecosystem, complementing systemic immunomodulation [6,8,9]. Oncolytic virotherapy is a promising approach addressing these limitations by direct tumor-selective infection with in situ immune activation [14].

Wild-type herpes simplex virus type 1 (HSV-1) is a naturally neurotropic double-stranded DNA virus with efficient cellular entry, rapid intracellular replication, and potent induction of immunogenic cell death through lytic infection. These intrinsic biological properties form the foundation of oncolytic herpes simplex virus (oHSV) therapy. However, wild-type HSV is unsuitable for clinical use because of its neurovirulence, unrestricted replication in normal neural tissue, and the risk of severe encephalitis. Therefore, therapeutic oHSV platforms are generated through rational attenuation of virulence while preserving tumor-selective replication. The most common modifications include deletion of γ34.5 (ICP34.5) and ICP6 (UL39) genes, reducing neurovirulence and restricting productive replication in normal brain tissue [14,15]. Importantly, the therapeutic impact of oHSV extends beyond direct oncolysis, as accumulating evidence indicates that oHSV-mediated immune modulation shapes the tumor microenvironment and critically influences therapeutic response [15,16,17].

Despite rapid progress, the immunological mechanisms by which oHSV influence the glioblastoma microenvironment remain fragmented across diverse experimental systems, viral constructs, and study designs. A comprehensive synthesis focused specifically on microenvironmental modulation, rather than on viral design or clinical safety alone, is therefore needed to identify unifying principles that govern therapeutic success and failure. Understanding how immune, stromal, and vascular compartments respond to oHSV infection is essential for the rational design of next-generation vectors and for the development of personalized clinical strategies.

This systematic review synthesizes preclinical and clinical evidence published between 2016 and 2025 to identify convergent mechanisms of tumor microenvironment reprogramming induced by oHSV therapy and to define key translational barriers that may limit its clinical efficacy in glioblastoma. Previous reviews have largely framed oHSV as a cytolytic or immunostimulatory modality; however, they have not captured the dynamic interplay between immune activation and therapy-induced resistance across tumor microenvironment compartments. By integrating findings across immune, stromal, and tumor-intrinsic pathways, this work proposes a conceptual framework in which oHSV functions as a temporally structured microenvironmental reprogramming system, providing a conceptual basis for the rational design of next-generation therapeutic strategies. This interpretation should be viewed primarily as an emerging hypothesis derived largely from preclinical evidence rather than as a clinically validated therapeutic model.

## 2. Materials and Methods

### 2.1. Study Design and Reporting Guidelines

This work was performed as a systematic review examining the immunomodulatory impact of oncolytic herpes simplex virus-based therapies on the glioblastoma tumor microenvironment. The review was designed and reported in accordance with the Preferred Reporting Items for Systematic Reviews and Meta-Analyses (PRISMA) guidelines and was prospectively registered in the International Prospective Register of Systematic Reviews (PROSPERO; registration number CRD420261295520).

### 2.2. Literature Search Strategy

A comprehensive and systematic literature search was performed in two electronic databases: PubMed (MEDLINE) and Embase (Elsevier). The final searches were conducted on 28 January 2026.

Search strategies were developed a priori and tailored separately for each database using a combination of controlled vocabulary terms (Medical Subject Headings MeSH for PubMed and Emtree terms for Embase) and free-text keywords. The strategies were intentionally restricted to oncolytic herpes simplex virus-based approaches to improve specificity and ensure alignment with the focused scope of the review.

The complete search strategies for both databases are provided in Table 1 to allow full reproducibility.

### 2.3. Eligibility Criteria

#### 2.3.1. Inclusion Criteria

Studies were included if they met all of the following criteria:

- focused on glioblastoma;
- investigated oncolytic herpes simplex virus (oHSV)-based therapies;
- addressed tumor microenvironment modulation, immunosuppression, or immune-related mechanisms;
- were original research articles (preclinical or clinical studies) published between 2016 and 2025.

#### 2.3.2. Exclusion Criteria

Studies were excluded if they:

- did not involve glioblastoma;
- investigated non-herpes simplex oncolytic viruses;
- were reviews, editorials, conference abstracts, letters, or commentaries;
- lacked mechanistic or immunological relevance to the tumor microenvironment.

### 2.4. Study Selection

Duplicate records were identified and removed manually. Study selection was performed in two sequential stages: title and abstract screening, followed by full-text assessment for eligibility. Both stages were conducted independently by two reviewers. Discrepancies were resolved through discussion with a third reviewer and consensus. A PRISMA flow diagram was generated to document the study selection process.

### 2.5. Data Extraction

Data extraction was performed independently by two reviewers using a predefined extraction framework. Extracted variables included:

- study design and model (in vitro, in vivo, or clinical);
- characteristics of the oHSV construct;
- mode of administration;
- effects on the tumor immune microenvironment;
- key immunological outcomes;
- major findings and limitations.

Any disagreements were discussed with a third reviewer and resolved by consensus. When information was incomplete or unclear, data were interpreted based on the descriptions provided in the original articles, and no additional assumptions were made beyond the reported results.

### 2.6. Data Synthesis

Due to substantial heterogeneity in study design, experimental models, viral constructs, and reported outcome measures, quantitative meta-analysis was not considered appropriate. Therefore, a qualitative narrative synthesis was performed to integrate findings across the included studies. All studies meeting the predefined eligibility criteria were included in the synthesis. Key outcomes related to tumor microenvironment modulation, including immune cell infiltration, cytokine signaling, macrophage polarization, adaptive immune activation, and mechanisms of therapeutic resistance, were analyzed descriptively across studies. Study characteristics and principal findings were summarized in tabular form to facilitate comparison. Because standardized effect estimates and summary statistics were not consistently available across studies, no statistical data transformations or effect size calculations were performed, and formal analyses of heterogeneity or sensitivity were not conducted.

### 2.7. Risk of Bias Assessment

Risk of bias was assessed qualitatively to evaluate the methodological robustness of the included studies. A structured qualitative quality assessment framework was applied, evaluating studies across predefined domains: model relevance (in vitro vs. in vivo vs. clinical), use of appropriate controls, reproducibility of experimental design, and completeness of outcome reporting. Two reviewers independently assessed the studies, and discrepancies were resolved through discussion and consensus. Because no quantitative synthesis or meta-analysis was performed, formal assessment of reporting bias due to missing results was not conducted.

## 3. Results

**Figure 1:**
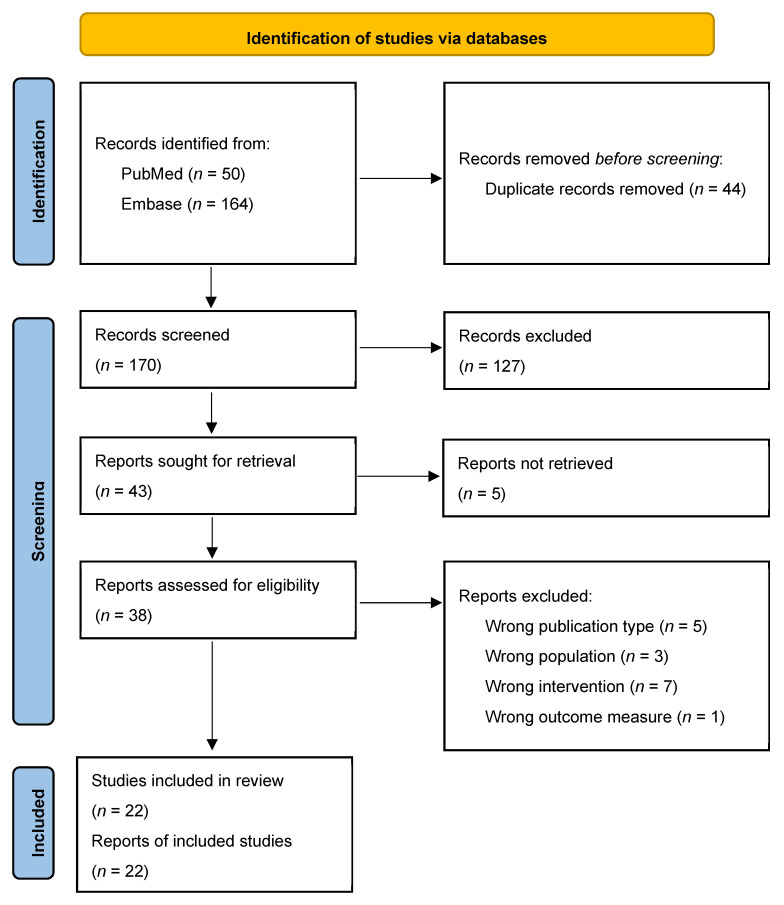
PRISMA 2020 [18]. Flow diagram summarizing the performed systematic review.

The initial literature search identified 214 records across the selected databases. After removal of duplicates, 170 unique articles remained for further evaluation. Title and abstract screening excluded studies that did not meet the predefined inclusion criteria, resulting in 38 articles eligible for full-text assessment. Following full-text review, 22 studies were ultimately included in the qualitative synthesis. The complete study selection process is summarized in a PRISMA flow chart (Figure 1), illustrating each stage of screening and eligibility assessment. The main reasons for exclusion at the full-text stage included studies not focusing on glioblastoma, investigations of non-herpes oncolytic viruses, review articles, and studies lacking mechanistic analysis of the tumor microenvironment.

Table 2 summarizes the structured qualitative assessment of methodological quality across the included studies based on predefined domains, including study type, use of appropriate controls, reproducibility of experimental design, and completeness of outcome reporting. Overall, included studies demonstrated adequate use of control groups and reported key outcomes clearly. Preclinical studies constituted the majority of the evidence–it supports cautious interpretation of the overall synthesis.

### 3.1. Cytokine-Based Reprogramming of the Immune Microenvironment

Arming oncolytic herpes simplex virus with interleukin-12 (IL-12) represents one of the most extensively validated strategies for overcoming the profound immunosuppression of the glioblastoma tumor microenvironment. The translational potential of this strategy was confirmed by Jackson et al., who directly compared unarmed and IL-12-armed oHSV in immunocompetent syngeneic mice glioblastoma models and demonstrated that IL-12 expression converts biologically inactive viral therapy into a durable immunotherapy [19]. While unarmed oHSV failed to improve survival, oHSV:IL-12 induced sustained macrophage and T-cell accumulation, expansion of T-cell receptor clonotypes, and long-term survival benefits. Single-cell transcriptomic analyses confirmed profound remodeling of the myeloid compartment, providing molecular evidence that IL-12 is a dominant driver of immune reprogramming rather than a secondary enhancer. In another study, Grimes et al. improved the understanding of how IL-12-armed oHSV shapes durable immune memory, suggesting that long-term tumor control after oHSV-IL12 therapy is critically dependent on CD4^+^ T cells rather than solely on cytotoxic CD8^+^ T cells [20]. oHSV-mediated reprogramming induced MHC class II expression on tumor and myeloid cells, enabling sustained CD4^+^ T-cell activation and the formation of memory populations regulated through an IL6Rα-Bcl-6 axis. This work revealed that IL-12 does not merely amplify immune responses but rewires antigen presentation and T-cell differentiation programs, indicating that CD4^+^ T-cell-centered memory circuit may be essential for durable tumor surveillance.

A series of human and murine glioblastoma stem-like cell models showed that combining IL-12-armed oHSV with the VEGFR (Vascular Endothelial Growth Factor Receptor) inhibitor axitinib produced superior antitumor efficacy compared with either monotherapy [21]. This effect reflected dual targeting of tumor vasculature and immune suppression, with reduced vascular density, increased macrophage infiltration, and extensive necrosis in human xenografts, while therapeutic benefit in syngeneic models was strictly dependent on an intact adaptive immune system. These findings reinforced the concept that IL-12-mediated immune activation is necessary but not always sufficient, and that full microenvironmental reprogramming may require concurrent disruption of stromal and vascular support. Further investigations suggested that the triple combination of IL-12-armed oHSV with anti-PD-1 and anti-CTLA-4 therapies eradicated tumors in the majority of treated glioblastoma mice [22]. Therapeutic cure was accompanied by a pronounced influx of macrophages and their polarization toward an M1-like, proinflammatory phenotype, together with a marked increase in the effector T cell to regulatory T cell ratio. Functional depletion experiments confirmed that macrophages were not merely bystanders but essential mediators of synergy, operating in concert with CD4^+^ and CD8^+^ T cells to sustain tumor eradication.

Complementary to IL-12-based approach, Bommareddy et al. engineered oHSV to express interleukin-2, a cytokine with well-established T-cell-stimulating properties but prohibitive systemic toxicity when administered systemically [23]. Local intratumoral delivery of IL-2 via oHSV significantly prolonged survival in immunocompetent glioblastoma models without detectable systemic adverse effects, suggesting that spatial confinement of IL-2 expression is sufficient to drive therapeutic immunity. The antitumor effect was associated with increased infiltration of cytotoxic CD8^+^ T cells and was strictly dependent on the presence of CD4^+^ helper T cells, indicating that IL-2 primarily reinforces coordinated T-cell responses rather than acting as a nonspecific immune stimulant. Notably, the lack of efficacy in poorly permissive tumor models underscores that cytokine-driven immune activation remains constrained by viral tropism and tumor-intrinsic resistance mechanisms.

### 3.2. Immune Consequences of oHSV Infection: Immunogenic Potential and Myeloid Resistance

Oncolytic herpes simplex virus does not require exogenous cytokine arming to exert profound immunomodulatory effects; instead, viral infection itself can generate and spatially confine potent immune mediators within the tumor niche. oHSV infection induces robust local release of interferon-γ (IFN-γ), primarily from infected tumor cells, which can be exploited to amplify antitumor immunity and enhance adoptive cellular immunotherapies [24]. In glioblastoma models, virus-induced IFN-γ increases infiltration of effector T cells, promotes recruitment of natural killer cells, and suppresses immunosuppressive mediators such as transforming growth factor β1 (TGF-β1), thereby converting the tumor microenvironment into a permissive niche for subsequent interventions, including CD70-specific chimeric antigen receptor (CAR) T cells. The resulting synergy indicates IFN-γ as a central mechanistic bridge linking viral oncolysis to amplification of adaptive immune effector functions.

Under permissive conditions, oHSV monotherapy can initiate a complete and durable cancer-immunity cycle. A histopathological analysis in mice demonstrated that the single intratumoral injection of an attenuated, unarmed oHSV-1 can generate long-term tumor control and immunological memory, as evidenced by rejection of tumor rechallenge in distant brain regions [25]. Durable protection was associated with extensive remodeling of the tumor microenvironment, including infiltration of CD4^+^ and CD8^+^ effector T cells, activation and redistribution of macrophages, microglia, and astroglia, marked disruption of the intratumoral vascular architecture, and the formation of intense fibrotic tissue consistent with complete tumor rejection. The residual tumor bed in long-term survivor mice was largely replaced by fibrous cells and showed absence of Ki67-positive proliferating tumor cells, indicating effective elimination of viable tumor tissue. MHC-II^+^ myeloid cells were also observed within peritumoral vessels and throughout the fibrotic tumor bed, together with prominent CD11b^+^ and CD68^+^ macrophage infiltration, suggesting profound restructuring of the tumor-supporting stromal and vascular niche [25]. Such remodeling may reduce nutrient supply and limit recurrence, but may also decrease subsequent penetration of systemically delivered therapies by replacing highly permeable tumor vasculature with dense fibrotic tissue. Therefore, therapies relying on vascular access may be most effective during the earlier inflammatory phase of oHSV treatment, before complete fibrotic consolidation occurs. The late fibrotic remodeling may represent a histological marker of durable local tumor control.

The described findings suggest that viral oncolysis alone can be sufficient to trigger long-lasting adaptive immunity when appropriate microenvironmental conditions are met. However, the immunogenic potential of oHSV is highly dependent on sustained virus-tumor interactions. Comparative analyses of clinically relevant oHSV-1 vectors indicate that although initial immune cell recruitment occurs regardless of vector, only tumors that maintain prolonged viral presence exhibit sustained accumulation of macrophages and CD4^+^ and CD8^+^ T cells, delayed tumor progression, and long-term survival [26]. In contrast, rapid viral clearance results in reversion of the tumor microenvironment to an immunologically quiescent state and complete therapeutic resistance, underscoring that oHSV-induced immune activation is transient unless continuously reinforced.

At the same time, virus-driven remodeling of the tumor microenvironment can activate counter-regulatory programs that limit therapeutic efficacy. oHSV infection can induce activation of NOTCH signaling within glioblastoma, leading to recruitment and expansion of immunosuppressive myeloid populations [27]. This process involves upregulation of the NOTCH ligand Jagged1 in infiltrating macrophages, amplifying NOTCH signaling across the tumor niche. NOTCH-activated macrophages secrete high levels of CCL2, promoting accumulation of myeloid-derived suppressor cells and reinforcing an immunosuppressive state that attenuates T-cell-mediated antitumor immunity. Clinically, translational relevance was supported by direct serum analysis from 18 patients with recurrent glioblastoma treated with the oHSV construct rQnestin34.5 in the phase I trial (NCT03152318) [27]. Study demonstrated that oHSV therapy induced sustained elevation of serum two key mediators of myeloid-driven immunosuppression-CCL2 and interleukin 10 (IL-10). Specifically, 9 of 18 patients showed a predefined increase in CCL2 for a minimum of two consecutive post-treatment time points. Similarly, sustained IL-10 elevation was observed in 9 of 18 patients, and 7 of these overlapped with the increased CCL2, supporting coordinated activation of an immunosuppressive myeloid program. These findings suggest that serum CCL2 and IL-10 may serve as clinically actionable biomarkers of incomplete immune conversion after oHSV therapy and support future combination strategies integrating NOTCH inhibition to overcome therapy-induced myeloid resistance.

Additional myeloid-mediated resistance programs are also induced by oHSV infection. Transcriptomic profiling has identified insulin-like growth factor 2 (IGF2) as a therapy-induced mediator that promotes recruitment of neutrophils and polymorphonuclear myeloid-derived suppressor cells (PMN-MDSCs), while enhancing secretion of immunosuppressive and proangiogenic cytokines [28]. Increased IGF2 expression has been detected in recurrent tumors from oHSV-treated patients, confirming clinical relevance. Engineering oHSV vectors to neutralize IGF2 signaling effectively disrupts this feedback loop, reduces suppressive myeloid infiltration, restores CD8^+^ T-cell recruitment, and significantly improves survival in intracranial tumor models, illustrating that virus-induced resistance pathways can be rationally intercepted through vector redesign. Finally, myeloid cells can also function as conduits of systemic antitumor immunity when appropriately instructed by the virus. In macrophage-dominant glioblastoma models, oHSV vectors engineered to express immune-activating ligands enable immune-mediated suppression of distant, noninfected tumor regions despite the absence of viral spread [29]. This abscopal-like effect is associated with accumulation of activated macrophages and T cells at distant sites and with upregulation of antigen processing and presentation pathways in myeloid cells. Notably, this response is potentiated by PD-1 blockade, whereas checkpoint inhibition alone is ineffective. Together, these findings indicate myeloid cells as a central regulatory axis in oHSV therapy-capable of sustaining immunosuppression or propagating systemic antitumor immunity depending on the molecular cues provided by the virus.

### 3.3. Overcoming Innate Immune Barriers and Antiviral Resistance

#### 3.3.1. NK Cell-Virus Interactions

Innate immune responses, particularly those mediated by natural killer (NK) cells, represent a double-edged sword in oncolytic herpes simplex virus therapy. While NK cells contribute to antitumor cytotoxicity, they also mediate rapid clearance of infected cells, thereby limiting viral spread and curtailing the duration of immune priming. Several studies have therefore focused on selectively modulating NK cell-virus interactions to preserve viral persistence while maintaining or redirecting innate antitumor activity. Xu et al. addressed this challenge by engineering an oHSV to express E-cadherin, a ligand for the inhibitory NK receptor KLRG1, thereby attenuating NK cell-mediated clearance of infected tumor cells without globally suppressing innate immunity [30]. Infection with this modified virus (OV-CDH1) induced ectopic E-cadherin expression on glioblastoma cells, enhancing cell–cell adhesion and facilitating intratumoral viral spread. At the same time, engagement of KLRG1 on NK cells reduced killing of virus-infected cells, prolonging viral persistence within the tumor. In vivo, this strategy significantly improved survival in glioblastoma-bearing mice, with therapeutic benefit primarily driven by enhanced intratumoral viral dissemination rather than direct inhibition of NK cell function. This work demonstrated that altering tumor cell-intrinsic adhesion and immune ligand expression can indirectly reprogram innate immune-virus dynamics, providing a generalizable approach to overcoming premature viral clearance. In contrast to strategies aimed at restraining NK-mediated antiviral activity, NK cells can be deliberately harnessed as dominant effectors of tumor clearance when appropriately redirected [31]. An oHSV expressing a full-length anti-CD47 antibody blocks the CD47-SIRPα “don’t eat me” signal locally within the tumor microenvironment, thereby enhancing innate immune recognition of tumor cells. The IgG1-expressing virus in particular induced potent antibody-dependent cellular cytotoxicity by NK cells and antibody-dependent phagocytosis by macrophages, leading to significantly improved survival compared with control oHSV or locally administered antibody. Importantly, antibody secretion was confined to the tumor microenvironment, avoiding systemic toxicity and enabling sustained engagement of innate effector cells.

#### 3.3.2. Multilevel Barriers to Oncolytic Virus Propagation

In addition to cellular immune effector mechanisms, glioblastoma harbors tumor-intrinsic and microenvironmental signaling networks that actively suppress oncolytic virus transduction, replication, and therapeutic propagation. A tumor-intrinsic antiviral resistance state was elucidated by Monie et al., who identified cellular communication network factor 1 (CCN1) as a central orchestrator of resistance to HSV-1-derived oncolytic immunotherapies [32]. Network-based analyses of transcriptomic data revealed that CCN1-high glioblastoma cells adopt a distinct intracellular signaling configuration enriched in innate antiviral response programs and cytokine-mediated signaling pathways that suppress viral transduction prior to productive infection. This antiviral state was characterized by dependency on specific regulatory nodes, including IDH1, TP53, and the protein homeostasis regulators RPL6, HUWE1, and COPS5. Importantly, this network architecture was conserved across large independent datasets of GBM cell lines and patient tumors, indicating that CCN1-driven resistance represents a common and clinically relevant barrier. These findings suggest that oHSV failure in a subset of tumors may be predetermined by tumor-intrinsic antiviral wiring, rather than by inadequate viral design or immune suppression alone.

Beyond tumor-intrinsic resistance, oHSV therapy can trigger microenvironmental damage responses that indirectly limit efficacy through vascular dysfunction and inflammatory toxicity. Hong et al. demonstrated that oHSV infection induces release of the damage-associated molecular pattern (DAMP) HMGB1 (High mobility group box 1), which in turn promotes endothelial permeability, vascular leakiness, and the formation of peritumoral edema [33]. Although HMGB1-driven inflammation is classically associated with immune activation, in this context it produced a deleterious microenvironment characterized by edema, impaired tissue integrity, and reduced therapeutic tolerance. Antibody-mediated neutralization of HMGB1 mitigated vascular leakage and significantly improved survival, identifying DAMP-driven vascular dysfunction as a previously underappreciated form of antiviral resistance that limits the therapeutic window of oHSV. Building on these observations, Swanner et al. directly targeted the endothelial response to oHSV-induced HMGB1 signaling by engineering an oHSV expressing the soluble decoy receptor for RAGE (Receptor for advanced glycation end products) ligands, thereby blocking RAGE-mediated endothelial activation [34]. This strategy reduced endothelial migration and activation, decreased vessel leakiness, and improved viral replication and tumor cell killing in co-culture systems. In vivo the strategy significantly enhanced therapeutic efficacy in orthotopic glioblastoma models, suggesting that endothelial cells represent an active antiviral barrier whose modulation can stabilize viral propagation and amplify antitumor effects. Together, these studies reveal that antiviral resistance is not limited to immune cells or tumor-intrinsic antiviral programs but extends to vascular and stromal components of the tumor microenvironment that respond maladaptively to viral infection.

#### 3.3.3. oHSV as a Delivery Platform for Immune Biologics

One of the most compelling extensions of oHSV-based immunotherapy is its use as a localized delivery platform for immune biologics, enabling high intratumoral concentrations of immunomodulatory agents while minimizing systemic exposure and toxicity. This strategy directly addresses one of the major limitations of systemic immune checkpoint inhibitors in glioblastoma, namely poor tumor penetration and dose-limiting immune-related adverse events, while leveraging virus-induced inflammation to create a receptive immunological niche. Proof of principle for this approach was provided by Passaro et al., who engineered an oncolytic herpes simplex virus to express and secrete a single-chain variable fragment antibody targeting PD-1 (oHSVscFvPD-1) [35]. This single-agent construct simultaneously preserved the lytic and immunogenic properties of oHSV while delivering local immune checkpoint blockade directly within the tumor microenvironment. In orthotopic glioma models, oHSVscFvPD-1 significantly prolonged survival compared with parental virus and generated a higher proportion of long-term survivors, many of which rejected tumor rechallenge, indicating durable immune memory. These findings suggest that checkpoint inhibition does not need to be systemically administered to achieve functional immune reprogramming and established oHSV as a viable vector for intratumoral biologic delivery.

Biologic designs have leveraged oHSV to spatially coordinate immune cell recruitment and tumor targeting. Tian et al. engineered a virus expressing a cetuximab-CCL5 fusion protein that tethered chemokine activity to EGFR-positive tumor cells, thereby generating a localized chemokine gradient that recruited NK cells, macrophages, and T cells while simultaneously inhibiting EGFR signaling [36]. This multifunctional construct integrated tumor targeting, immune cell recruitment, and signal inhibition into a single virally encoded biologic, resulting in robust tumor regression and prolonged survival in immunocompetent models. The study exemplifies how oHSV can be used to impose spatial control over immune modulation, a critical requirement in the highly sensitive central nervous system environment.

The capacity of oHSV to deliver T-cell-redirecting biologics was demonstrated by Baugh et al., who incorporated a bispecific T-cell engager targeting CD3 and NKG2D ligands into the oHSV [37]. Local secretion of the BiTE enabled antigen-independent T-cell activation and killing of both infected tumor cells and non-permissive glioma stem-like cells, effectively extending the reach of virotherapy beyond cells directly susceptible to viral infection. Importantly, DNA damage induced by temozolomide and radiation further increased NKG2D ligand expression, creating a rational synergy between conventional therapy and virus-delivered biologics.

Finally, Akl et al. expanded the scope of oHSV-based biologic delivery beyond tumor cells to non-malignant components of the microenvironment by targeting astrocyte-driven immunosuppression [38]. By engineering oHSV to express a TRAIL-blocking single-chain antibody, the authors neutralized astrocyte-mediated T-cell apoptosis driven by IL-11-STAT3 signaling, restoring tumor-specific immunity and prolonging survival in preclinical models. This work suggests that oHSV can be used as a precision tool to reprogram stromal and neural elements of the tumor niche, not merely immune or malignant cells.

#### 3.3.4. Clinical Translation and Impact of Clinical Modifiers on oHSV Efficacy

The progressive refinement of oncolytic herpes simplex virus platforms has culminated in a growing body of evidence demonstrating that the immunological mechanisms identified in preclinical models are not merely experimental phenomena but can translate into meaningful clinical effects in patients with glioblastoma. The phase I CAN-3110 trial directly linked intratumoral oHSV-induced immune activation to survival benefit in recurrent glioblastoma [39]. In this study, intralesional administration of a nestin-restricted, ICP34.5-retaining oHSV was safe and resulted in preferential tumor replication without dose-limiting toxicities. Importantly, survival benefit was not correlated with viral dose or persistence alone but instead with immune activation signatures, including increased intratumoral and peripheral T-cell infiltration, dynamic remodeling of T-cell clonotypes, and tumor transcriptomic programs consistent with immune engagement.

HSV-1 seropositivity was one of the most important clinical determinants of the durable remodeling observed after CAN-3110 therapy. In HSV-1-seropositive patients, durable remodeling was characterized by sustained intratumoral CD4^+^ and CD8^+^ T-cell infiltration, greater TCRβ (T-cell receptor beta) diversity and clonal expansion, persistent antitumor cytokine and immune activation signatures, and significantly prolonged survival compared with seronegative patients-median overall survival in GBM was 14.2 months in HSV-1-seropositive patients versus only 7.8 months in seronegative patients (*p* = 0.007) [39]. Importantly, HSV-1 seropositivity was also confirmed as an independent predictor of survival on multivariate Cox analysis, stronger than many conventional clinical variables. At the same time, a rapid pre-existing antiviral memory response in HSV-1-seropositive patients resulted in viral clearance rather than prolonged viral persistence. The observation that HSV-1-seropositive patients exhibited superior survival and more robust immune activation provides a clinical evidence that antiviral immunity can be harnessed to amplify antitumor responses.

Microenvironmental conditions intrinsic to glioblastoma further modulate tumor susceptibility to oHSV, particularly within therapy-resistant stem-like compartments. Vazifehmand et al. indicated that the impact of HSV-G47Δ on telomere biology in glioblastoma stem cells is highly dependent on oxygen tension [40]. Under hypoxic conditions, which characterize invasive and treatment-resistant tumor niches, oHSV exposure induced telomere shortening and dysregulation of telomerase and DNA repair pathways, suggesting increased genomic instability and vulnerability. In contrast, normoxic conditions favored telomere elongation, highlighting the context-dependent nature of oHSV-induced molecular effects. These findings indicate that hypoxia may sensitize otherwise resistant glioblastoma stem cells to oHSV-mediated genomic disruption, adding a new dimension to understanding spatial heterogeneity in therapeutic response.

Table 3 summarizes the key characteristics of the 22 studies included in this systematic review, detailing the experimental or clinical design, glioblastoma models or patient populations, oHSV platforms and genetic modifications, routes of administration, combinatorial strategies, immunomodulatory and tumor microenvironment–related effects, and reported therapeutic or survival outcomes.

## 4. Discussion

**Figure 2:**
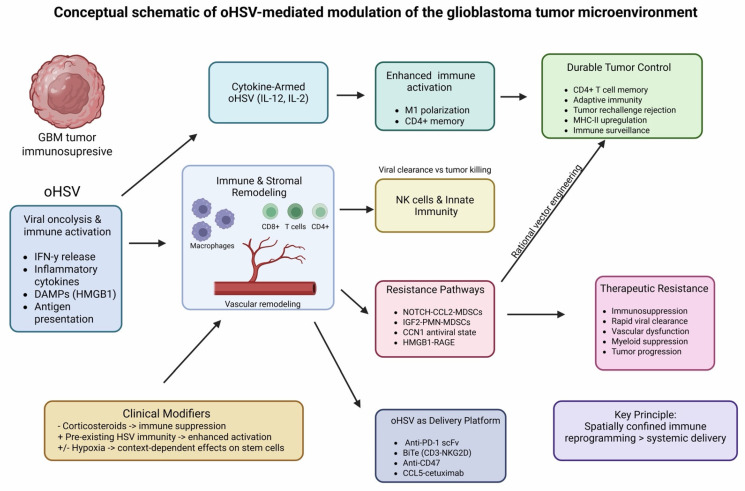
oHSV-mediated reprogramming of the glioblastoma tumor microenvironment. This figure represents a conceptual summary of findings derived predominantly from preclinical studies, supplemented by limited clinical observations, and should not be interpreted as a unified or clinically validated biological pathway. oHSV therapy initiates localized oncolysis and innate immune activation, characterized by interferon release, inflammatory cytokine production, DAMP signaling, and enhanced antigen presentation. These early events drive immune and stromal remodeling within the glioblastoma niche, including macrophage polarization, T-cell recruitment, and vascular reorganization. Cytokine-armed oHSV vectors (e.g., IL-12, IL-2) further amplify immune activation and support the development of adaptive immune memory, whereas innate immune responses, particularly natural killer (NK) cell activity, contribute to both viral clearance and tumor cell killing. Therapeutic outcomes are shaped by competing resistance pathways, including MDSC expansion, antiviral states, and stromal signaling circuits, which may limit durability of response. Elements within the schematic are annotated to reflect the level of supporting evidence, distinguishing findings derived from clinical versus preclinical studies. BiTE, bispecific T-cell engager; CCL2, C-C motif chemokine ligand 2; CCL5, C-C motif chemokine ligand 5; CCN1, cellular communication network factor 1 (CYR61); CD4+ T cells, CD4-positive helper T lymphocytes; CD8+ T cells, CD8-positive cytotoxic T lymphocytes; CD47, cluster of differentiation 47; DAMPs, danger-associated molecular patterns; GBM, glioblastoma; HMGB1, high-mobility group box 1 protein; HSV, herpes simplex virus; IFN-γ, interferon gamma; IGF2, insulin-like growth factor 2; IL-2, interleukin 2; IL-12, interleukin 12; MDSCs, myeloid-derived suppressor cells; MHC-II, major histocompatibility complex class II; NKG2D, natural killer group 2D receptor; NK, natural killer; NOTCH, Notch signaling pathway; oHSV, oncolytic herpes simplex virus; PD-1, programmed cell death protein 1; PMN-MDSCs, polymorphonuclear myeloid-derived suppressor cells; RAGE, receptor for advanced glycation end products; scFv, single-chain variable fragment; TME, tumor microenvironment. Created in https://BioRender.com.

The available evidence suggests that, in addition to direct oncolysis, oncolytic herpes simplex virus therapy exerts its therapeutic effects in glioblastoma largely through immune microenvironment reprogramming. The complex, spatially confined immunological effects of oHSV therapy on the glioblastoma microenvironment, including immune activation, stromal remodeling, and resistance pathways, are summarized in Figure 2. The model integrates findings across heterogeneous study types and should be interpreted as a conceptual framework rather than a definitive pathway.

Across predominantly preclinical studies and limited early clinical observations, therapeutic benefit often appears to correlate with the extent and quality of tumor microenvironment remodeling rather than with viral replication alone. oHSV initiates a cascade of spatially confined inflammatory signals that can convert the profoundly immunosuppressive glioblastoma niche into a transiently permissive immune ecosystem, thereby enabling adaptive immune engagement that is otherwise absent in this disease. These findings are directly supported by clinical observations from the CAN-3110 trial, where immune activation signatures predicted survival, providing rare clinical validation of immune-mediated efficacy in this tumor type.

A recurring observation across preclinical studies is the prominent role of adaptive immunity, particularly CD4^+^ T-cell-dependent immune orchestration, in sustaining long-term tumor control. In many preclinical studies, IL-12-armed oHSV vectors induced robust and durable responses, driving macrophage polarization, enhanced antigen presentation, and formation of long-lived immune memory. Data suggesting that durable tumor rejection depends on CD4^+^ T-cell programs rather than solely on cytotoxic CD8^+^ T cells fundamentally reframe the conceptual basis of viro-immunotherapy in glioblastoma.

Myeloid cells emerge as the dominant regulatory axis determining therapeutic success or failure. While oHSV-induced macrophage infiltration can mediate tumor eradication when appropriately instructed, the same compartment can rapidly re-establish immunosuppression through NOTCH-CCL2 or IGF2-driven feedback loops, leading to recruitment of suppressive myeloid populations and therapeutic resistance. Importantly, studies suggest that these resistance mechanisms are actively induced by therapy and can be intercepted through rational vector engineering. This highlights that next-generation oHSV platforms must be designed not only to activate immunity but also to preemptively block predictable counter-regulatory programs.

Innate immunity further shapes therapeutic outcomes by controlling viral persistence. Rapid NK cell-mediated clearance limits immune priming, whereas selective modulation of NK-virus interactions prolongs viral bioavailability without abolishing antitumor activity. These data emphasize that therapeutic efficacy requires a delicate balance between antiviral defense and sustained immune education, a balance that is particularly critical in the central nervous system where excessive inflammation carries significant risk.

Beyond immune cells, vascular and stromal components of the glioblastoma microenvironment constitute additional, often overlooked, barriers to effective virotherapy. HMGB1-mediated endothelial dysfunction, vascular leakage, and edema represent maladaptive host responses that restrict viral propagation and therapeutic tolerance. Successful targeting of these pathways through vectorized decoy receptors demonstrates that endothelial cells are active participants in antiviral resistance and must be considered in the design of clinically viable oHSV platforms.

A major translational advance highlighted in this review is the evolution of oHSV into a localized delivery system for immune biologics. Viral expression of checkpoint inhibitors, bispecific T-cell engagers, chemokine-antibody fusion proteins, and stromal modulators enables high intratumoral concentrations with minimal systemic toxicity, overcoming a central limitation of systemic immunotherapy in glioblastoma. These studies establish oHSV as a self-amplifying, spatially restricted immunotherapy factory, capable of coordinating immune activation, targeting, and persistence within the tumor niche.

Clinical translation is strongly modulated by patient-specific and treatment-related factors. Pre-existing antiviral immunity, historically viewed as detrimental, may enhance therapeutic responses by amplifying immune engagement when viral persistence is adequate. Microenvironmental conditions such as hypoxia further shape tumor susceptibility, particularly within therapy-resistant stem-like niches, underscoring the spatial heterogeneity of response.

An important conceptual distinction is that the therapeutic activity of oHSV originates from, but is not equivalent to, the native biology of wild-type HSV. Wild-type HSV possesses inherent oncolytic potential through rapid lytic replication, strong induction of danger-associated molecular patterns, and robust activation of innate antiviral immunity. In principle, these properties alone can generate direct tumor cytotoxicity and initiate local inflammatory conversion of an immunologically “cold” glioblastoma microenvironment. However, the studies included in this review consistently demonstrate that the intrinsic viral properties of oHSV are rarely sufficient to produce durable disease control in glioblastoma. Unarmed oHSV monotherapy can induce transient immune activation and, under favorable conditions, long-term immune memory, but therapeutic success is highly dependent on sustained viral persistence and permissive microenvironmental conditions. In many models, durable benefit required either cytokine arming, checkpoint modulation, stromal targeting, or interception of therapy-induced resistance pathways.

Collectively, the available evidence indicates that oHSV therapy is constrained not by insufficient immunogenic potential, but by the simultaneous activation of counter-regulatory processes across multiple levels of the tumor ecosystem. Effective treatment requires sustained viral persistence, coordinated activation of adaptive immunity, and prevention of therapy-induced resistance within myeloid, stromal, and vascular compartments. However, these processes are frequently misaligned in practice, as antiviral responses limit viral propagation, while compensatory immunosuppressive pathways and microenvironmental barriers restrict durable immune engagement. In addition, tumor-intrinsic resistance states and clinically relevant modifiers further contribute to variability in therapeutic response. Taken together, these factors highlight that oHSV efficacy depends on precise orchestration of interconnected biological systems, rather than on any single mechanism alone.

Despite encouraging mechanistic and early clinical findings, several translational challenges remain before oHSV can be broadly implemented in glioblastoma care. First, most available evidence derives from immunocompetent murine models, which only partially recapitulate the spatial, immunological, and therapeutic complexity of human GBM. Differences in immune system composition, tumor evolution, and microenvironmental architecture can substantially alter therapeutic responses. Second, substantial heterogeneity across viral backbones, transgene payloads, dosing schedules, and routes of administration complicates cross-study comparison and limits immediate standardization for clinical protocols. In addition, viral delivery methods in experimental settings often achieve more uniform intratumoral distribution than is feasible in clinical practice. Third, therapeutic activity appears highly context-dependent, being influenced by viral persistence, intratumoral immune composition, hypoxia, and treatment-related modifiers. In addition, practical issues such as optimal delivery techniques, timing relative to standard therapies, and maintaining sufficient viral activity within the tumor remain key challenges for clinical translation. Furthermore, patient populations are highly heterogeneous, and the absence of validated biomarkers for treatment selection may dilute potential therapeutic benefit.

The findings of this review support a conceptual shift in the development of oHSV-based therapies, from a predominantly oncolytic approach toward a strategy focused on immune microenvironment reprogramming. Future therapeutic designs should prioritize combination strategies that address both immune activation and resistance mechanisms, including integration with immune checkpoint inhibitors, targeted inhibition of myeloid-driven immunosuppression, and modulation of antiviral responses. The identification of predictive biomarkers, such as baseline immune infiltration, antiviral immune status, and tumor-specific resistance pathways, will be essential for optimizing patient selection and improving clinical outcomes. Advances in viral engineering further enable the incorporation of immunomodulatory transgenes and biologics, positioning oHSV as a multifunctional platform for localized immune modulation. Collectively, these approaches may enhance the likelihood of achieving durable clinical responses in glioblastoma. From a clinical perspective, the most realistic near-term application of oHSV is as a locoregional immunomodulatory platform for selected patients with recurrent glioblastoma, particularly in protocols integrating image-guided intratumoral delivery, careful management, and combination with complementary therapeutic approaches.

Rather than functioning primarily as a cytolytic agent, oHSV therapy can be conceptualized as a temporally structured microenvironmental reprogramming system. In this model, early innate activation is followed by a critical transition phase dominated by myeloid reprogramming, which determines whether adaptive immunity is sustained or suppressed. Therapeutic resistance is therefore not intrinsic but dynamically induced by the same pathways that initially enable immune activation. This framework suggests that effective oHSV-based therapies must be designed as sequential interventions targeting both activation and feedback inhibition phases of the tumor ecosystem.

Before interpreting the therapeutic implications of the findings, it is important to distinguish the strength of evidence across study types. The vast majority of included studies were preclinical investigations using orthotopic murine models, genetically engineered viral constructs, and highly controlled experimental conditions. These studies provide strong mechanistic insight into immune activation, myeloid remodeling, vascular responses, and resistance pathways, but their translational generalizability remains inherently limited. In contrast, direct clinical evidence remains restricted primarily to early-phase studies such as the CAN-3110 trial and small translational patient cohorts evaluating biomarkers of immune response. Therefore, conclusions regarding durable clinical efficacy, immune memory, and long-term tumor microenvironment reprogramming should be interpreted cautiously, with preclinical observations viewed as hypothesis-generating rather than definitive clinical proof.

## 5. Conclusions

Taken together, the available evidence supports the emerging interpretation that oHSV may function not only as an oncolytic agent but also as a programmable immunomodulatory platform capable of influencing multiple components of the glioblastoma microenvironment. However, this concept remains driven predominantly by preclinical evidence, and its clinical applicability requires further validation in prospective trials specifically designed to assess immune remodeling endpoints rather than safety or short-term radiographic response alone. Therapeutic success depends on sustained, coordinated reprogramming of immune, vascular, and stromal compartments, and on the rational interception of induced resistance pathways. These insights provide a mechanistic foundation for the design of next-generation multifunctional oHSV vectors and for personalized clinical strategies aimed at finally unlocking effective immunotherapy for glioblastoma.

## Figures and Tables

**Table 1:** Search strategy used for each database: Pubmed (MEDLINE), and Embase (Elsevier).

Database	Search Strategy
PubMed (MEDLINE)	(“Glioblastoma”[Mesh] OR glioblastoma[tiab] OR “glioblastoma multiforme”[tiab] OR GBM[tiab]) AND (“Oncolytic Viruses”[Mesh] OR “oncolytic virus”[tiab] OR “oncolytic virotherapy”[tiab] OR virotherapy[tiab]) AND (“Tumor Microenvironment”[Mesh] OR “Immunosuppression”[Mesh] OR microenvironment[tiab] OR immunosuppress*[tiab] OR immunomodulat*[tiab]) AND (“Simplexvirus”[Mesh] OR HSV-1[tiab] OR “herpes simplex virus”[tiab] OR “oncolytic herpes simplex virus”[tiab] OR oHSV[tiab] OR G47Δ[tiab] OR teserpaturev[tiab] OR rQNestin34.5[tiab])
Embase (Elsevier)	(‘glioblastoma’/exp OR glioblastoma:ti,ab OR ‘glioblastoma multiforme’:ti,ab OR GBM:ti,ab) AND (‘oncolytic virus’/exp OR ‘oncolytic virus’:ti,ab OR ‘oncolytic virotherapy’:ti,ab OR virotherapy:ti,ab) AND (‘tumor microenvironment’/exp OR ‘immunosuppression’/exp OR microenvironment:ti,ab OR immunosuppress*:ti,ab OR immunomodulat*:ti,ab) AND (‘simplexvirus’/exp OR HSV-1:ti,ab OR ‘herpes simplex virus’:ti,ab OR ‘oncolytic herpes simplex virus’:ti,ab OR oHSV:ti,ab OR G47Δ:ti,ab OR teserpaturev:ti,ab OR rQNestin34.5:ti,ab)

**Table 2:** Methodological quality assessment of included studies. A structured qualitative appraisal of the methodological rigor of all included studies, evaluated across four predefined domains: study design, use of appropriate controls, reproducibility of experimental design, and completeness of outcome reporting.

First Author (Year)	Study Design	Use of Appropriate Controls	Reproducibility of Experimental Design	Completeness of Outcome Reporting
Jackson (2025) [19]	Preclinical (in vivo + in vitro)	Includes appropriate vehicle and unarmed oHSV control groups	Methods clearly described (virus construction, animal procedures, flow cytometry, scRNA-seq, TCR-seq); multiple independent experiments reported	Comprehensive reporting of survival, immune profiling, and transcriptomic analyses
Grimes (2025) [20]	Preclinical (in vivo + in vitro)	Includes saline, isotype antibody, depletion controls, and parental virus comparisons	Detailed experimental design with replication across models and techniques	Comprehensive reporting including survival, flow cytometry, scRNA-seq, functional assays, and mechanistic validation (e.g., MHCII dependence, adoptive transfer)
Saha (2018) [21]	Preclinical (in vivo)	Includes vehicle, monotherapy, combination, and immune-depletion controls	Methods extensively described with replication and statistical analyses	Comprehensive reporting including survival, angiogenesis (CD34), immune infiltration, signaling pathways, and stemness assays
Saha (2017) [22]	Preclinical (in vivo + in vitro)	Includes multiple treatment arms and immune depletion experiments	Clearly described experimental design with multiple models	Comprehensive reporting including survival outcomes, immune cell infiltration (T cells, macrophages), polarization (M1-like), and mechanistic validation
Bommareddy (2024) [23]	Preclinical (in vivo + in vitro)	Includes PBS (vehicle) controls, parental tumor models, immunocompetent vs. athymic mice, and extensive immune cell depletion controls (CD4^+^, CD8^+^, NK, isotype antibodies)	Experimental design well described (engineered oHSV G47Δ-IL2, orthotopic GSC-derived GBM models, flow cytometry, IHC, survival studies, immune depletion experiments); multiple complementary models and replicates used	Comprehensive reporting including survival benefit, immune infiltration (CD3^+^, CD8^+^ T cells), cytokine effects, mechanistic immune dependency (critical role of CD4^+^ T cells), and validation across immunocompetent vs. immunodeficient systems
Zhu (2022) [24]	Preclinical (in vivo + in vitro)	Includes control T cells, oHSV-1 alone, CAR T alone, and combination groups	Detailed experimental workflow (CAR T construction, virus engineering, humanized mouse model, flow cytometry, ELISA, IVIS); multiple experiments and donors reported	Comprehensive reporting including cytokine profiles, immune cell infiltration (CD4^+^, CD8^+^, NK, Treg), tumor regression, and survival outcomes
Reale (2024) [25]	Preclinical (in vivo + in vitro)	Includes vehicle (PBS) control and untreated tumor-bearing groups	Experimental design well described (virus construction, in vitro assays, orthotopic model, randomization, power calculation, blinded IHC); multiple replicates reported	Comprehensive reporting including survival analysis, tumor rechallenge, immune infiltration (CD4^+^, CD8^+^, FOXP3^+^), myeloid/astroglial remodeling, and histopathological validation
Jackson (2021) [26]	Preclinical (in vivo + in vitro)	Includes vehicle (PBS) controls and comparison between different oHSV constructs	Experimental design described (virus engineering, orthotopic implantation, flow cytometry, immune profiling); multiple models used	Reporting includes survival, viral persistence, and immune infiltration; mechanistic depth more limited compared to transcriptomic studies
Otani (2022) [27]	Preclinical (in vivo + in vitro + translational component)	Includes vehicle (PBS/DMSO), oHSV-treated groups, and pharmacologic NOTCH inhibition (GSI); also uses genetic and antibody-based interventions	Experimental design highly detailed (RNA-seq, flow cytometry, in vivo survival models, co-culture systems, cytokine assays, TCGA analysis); multiple complementary approaches	Extensive reporting including immune infiltration, cytokine profiles, gene expression, survival, and translational patient serum data; mechanistic pathways thoroughly explored
Noh (2024) [28]	Preclinical (in vivo + in vitro + translational component)	Includes PBS controls, parental oHSV comparisons, and combination with immune checkpoint blockade; additional depletion experiments performed	Experimental design well described (viral engineering, orthotopic models, flow cytometry, cytokine assays, transcriptomics); multiple complementary approaches used	Comprehensive reporting including survival, immune infiltration (CD8^+^ T cells, neutrophils), cytokine profiling, and mechanistic validation (IGF2 axis)
Wirsching (2019) [29]	Preclinical (in vivo + in vitro)	Includes PBS controls, unarmed oHSV comparison, and combination with anti–PD-1; multiple comparator arms used	Detailed methodology including genetic model generation, virotherapy administration, flow cytometry, gene expression profiling (nCounter), and bilateral tumor design enabling mechanistic insights	Extensive reporting including survival, immune profiling (T cells, TAMs, NK cells), gene expression analyses (GO/KEGG), abscopal effects, and mechanistic immune pathways (TLR, antigen presentation)
Xu (2019) [30]	Preclinical (in vitro + in vivo)	Includes parental oHSV (OV-Q1), mutant virus (OV-IL2RA-CDH1), vehicle controls, NK cell subset analyses (KLRG1+/−), and immune cell depletion experiments (NK, macrophages, CD4^+^, CD8^+^)	Detailed experimental protocols including viral engineering, plaque assays, NK cytotoxicity assays, flow cytometry, in vivo intracranial models, survival analyses, and multiple independent replicates (often stated as ≥3 or ≥5 repeats)	Comprehensive reporting including viral spread, NK cell interactions, immune profiling, survival outcomes, viral kinetics, mechanistic assays (cell fusion, cadherin interaction), and safety/biodistribution data
Xu (2021) [31]	Preclinical (in vivo + in vitro)	Includes parental oHSV (OV-Q1), isotype antibody controls, IgG1 vs. IgG4 variants, and untreated controls	Methods clearly described (viral engineering, antibody expression, orthotopic implantation, flow cytometry, ADCP/ADCC assays); multiple experiments and comparative arms included	Comprehensive reporting including survival, immune cell infiltration (macrophages, NK cells), phagocytosis assays, antibody expression, and mechanistic comparisons between Fc variants
Monie (2021) [32]	Computational/in silico study	Includes comparison between CCN1-induced and control states across datasets; no experimental biological controls	Methods clearly described (network modeling using NetDecoder, integration of CCLE and TCGA datasets, enrichment analyses); reproducible computational workflow outlined	Comprehensive reporting including network analyses, pathway enrichment (KEGG, GO), gene dependency data, and validation across independent datasets; limitations of model explicitly discussed
Hong (2019) [33]	Preclinical (in vitro and in vivo)	Includes multiple controls: uninfected cells, PBS-treated animals, isotype antibody controls, and comparison with/without HMGB1 blockade	Experimental procedures well-described (MOI, ELISA, flow cytometry, IVIS, MRI, animal protocols), enabling reproducibility; multiple independent experiments reported	Extensive outcome reporting including HMGB1 secretion, endothelial activation, vascular permeability, viral replication, tumor growth, survival, and MRI-based edema assessment
Swanner (2023) [34]	Preclinical (in vitro and in vivo)	Includes multiple controls: PBS-treated groups, control virus (rHSVQ), isotype antibodies, HMGB1/RAGE blockade comparisons, uninfected controls	Detailed methodology provided (MOI, co-culture systems, ELISA, qPCR, flow cytometry, migration assays, in vivo stereotactic implantation), enabling reproducibility; experiments performed in replicates	Comprehensive reporting including endothelial activation, migration, permeability, viral replication, signaling pathways (pERK/pMEK), tumor growth, and survival outcomes
Passaro (2019) [35]	Preclinical (in vivo + in vitro)	Includes parental oHSV (NG34), untreated controls, anti–PD-1 antibody comparisons, and immunocompetent vs. athymic models to assess immune dependency	Detailed experimental design (viral engineering, ELISA, cytotoxicity assays, orthotopic intracranial models, survival analysis, qPCR); multiple independent experiments and complementary models reported	Comprehensive reporting including survival outcomes, tumor rechallenge (immune memory), viral kinetics, transgene expression (scFvPD-1), and mechanistic immune dependence (T-cell requirement)
Tian (2022) [36]	Preclinical (in vivo + in vitro + translational component)	Includes parental oHSV (OV-Q1), saline controls, fusion protein controls (Cmab-CCL5 vs. isotype), EGFR− tumor controls, and immune cell depletion (NK, macrophages, T cells)	Highly detailed experimental design (viral engineering, fusion protein validation, ELISA, flow cytometry, ADCC/ADCP assays, orthotopic and xenograft GBM models, immune reconstitution, depletion studies); multiple complementary systems and replicates used	Extensive reporting including tumor growth, survival (multiple models and treatment cycles), immune cell migration, activation (NK, macrophages, T cells), cytokine profiling, ADCC/ADCP mechanisms, abscopal effects, and signaling pathway modulation (EGFR/AKT)
Baugh (2024) [37]	Preclinical (in vivo + in vitro)	Includes parental oHSV (G207), uninfected/untreated controls, TMZ/radiation conditions, and BiTE vs. non-BiTE comparisons to isolate therapeutic contribution	Experimental design clearly described (viral engineering of G207-NKG2D BiTE, co-culture assays with T cells, GBM stem-like cell models, orthotopic xenograft systems); multiple complementary approaches used to validate mechanism	Comprehensive reporting including T-cell activation (CD3-mediated), cytotoxicity against GBM and GSCs, interaction with standard-of-care therapies (TMZ/radiation), and mechanistic insights into NKG2DL targeting and immune redirection
Akl (2025) [38]	Preclinical (in vivo + in vitro + translational component)	Includes multiple controls: untreated/PBS, non-targeting sgRNA controls, TRAIL/IL-11 pathway genetic perturbations (astrocyte-specific knockouts), control viruses (empty and nonspecific scFv), and T cell–deficient models (Rag2−/−)	Highly detailed and multimodal design (scRNA-seq, snRNA-seq, spatial transcriptomics, CRISPR perturbations, orthotopic GBM models, flow cytometry, co-culture assays, RNA-seq); extensive use of complementary systems and validation across mouse and human samples	Extensive reporting including survival, T cell apoptosis and activation, immune infiltration (CD4^+^, CD8^+^, TAMs), cytokine profiling, mechanistic IL-11–STAT3–TRAIL axis validation, spatial and transcriptomic analyses, and therapeutic efficacy of engineered oHSV targeting TRAIL pathway
Ling (2023) [39]	Clinical (phase I trial with translational analyses)	No traditional experimental controls; internal comparisons include HSV1 seropositive vs. seronegative patients, pre- vs. post-treatment tumor samples, and longitudinal PBMC analyses	Methods well described (clinical protocol, dosing cohorts, MRI-guided delivery, immunohistochemistry, TCR sequencing, RNA-seq, statistical analyses); reproducible clinical and translational workflow outlined	Comprehensive reporting including safety (no dose-limiting toxicity), survival (median OS, subgroup analyses), immune infiltration (CD4^+^/CD8^+^ TILs), TCR clonality/diversity, viral persistence, and transcriptomic immune activation signatures correlated with outcomes
Vazifehmand (2024) [40]	Preclinical (in vitro + in vivo)	Includes parental oHSV controls, BiTE-negative virus comparisons, uninfected controls, and functional comparisons of PD-L1 targeting vs. baseline conditions	Experimental design clearly described (engineering of PD-L1–targeting BiTE-expressing oHSV, co-culture assays, cytotoxicity assays, orthotopic tumor models, immune profiling); multiple complementary assays and replicates used	Comprehensive reporting including T-cell activation, tumor cell killing, immune synapse formation, cytokine release, and in vivo tumor control; mechanistic validation of PD-L1–directed immune redirection

**Table 3:** Characteristics of studies included in the systematic review (*n* = 22). ↑, increase/upregulation/enhancement compared to the respective control/comparator group; ↓, decrease/downregulation/reduction compared to the respective control/comparator group; ADCC, antibody-dependent cellular cytotoxicity; ADCP, antibody-dependent cellular phagocytosis; BCBM, brain cancer brain metastasis; BiTE, bispecific T-cell engager; CAR, chimeric antigen receptor; CCL, C-C motif chemokine ligand; CMV, cytomegalovirus promoter; CTLA-4, cytotoxic T-lymphocyte–associated protein 4; DC, dendritic cell; DKC1, dyskerin pseudouridine synthase 1; EGFR, epidermal growth factor receptor; ERK, extracellular signal-regulated kinase; Fc, fragment crystallizable region; FFL, firefly luciferase; GBM, glioblastoma; GBMCSC, glioblastoma cancer stem cell; GEMM, genetically engineered mouse model; GSC, glioma stem cell; GSI, gamma-secretase inhibitor; GzmB, granzyme B; HMGB1, high-mobility group box 1; hTERC, human telomerase RNA component; HSV, herpes simplex virus; ICAM-1, intercellular adhesion molecule-1; ICP, infected cell protein; ICB, immune checkpoint blockade; IDH, isocitrate dehydrogenase; IE, immediate-early promoter; IFN-γ, interferon gamma; IGF, insulin-like growth factor; IGF1R, insulin-like growth factor 1 receptor; IGF2R, insulin-like growth factor 2 receptor; IL, interleukin; iMRI, intraoperative magnetic resonance imaging; IP, intraperitoneal; KLRG1, killer cell lectin-like receptor subfamily G member 1; LTS, long-term survivor; MDSC, myeloid-derived suppressor cell; MGMT, O-6-methylguanine-DNA methyltransferase; MHC, major histocompatibility complex; MOI, multiplicity of infection; MRN, MRE11–RAD50–NBS1 DNA repair complex; MRI, magnetic resonance imaging; mOS, median overall survival; NK, natural killer (cell); NSG, NOD scid gamma mouse; oHSV, oncolytic herpes simplex virus; OS, overall survival; PBS, phosphate-buffered saline; PD-1, programmed cell death protein 1; PD-L1, programmed death-ligand 1; PDGF, platelet-derived growth factor; PFU, plaque-forming unit; PMN-MDSC, polymorphonuclear myeloid-derived suppressor cell; PFS, progression-free survival; RAGE, receptor for advanced glycation end products; RT, radiotherapy; scFv, single-chain variable fragment; STAT, signal transducer and activator of transcription; TAM, tumor-associated macrophage; TCGA, The Cancer Genome Atlas; TCR, T-cell receptor; TGF-β, transforming growth factor beta; TH1, T helper 1; TIL, tumor-infiltrating lymphocyte; TK, thymidine kinase; TME, tumor microenvironment; TNF, tumor necrosis factor; TRAIL, TNF-related apoptosis-inducing ligand; VEGFR, vascular endothelial growth factor receptor.

First Author (Year)	Model/Population	oHSV Strain	Genetic Modifications	Route of Administration	Combination Therapy	Primary Immunomodulatory Effects	Tumor Microenvironment Changes	Survival/Efficacy Outcomes	Key Conclusions
Jackson (2025) [19]	Murine orthotopic syngeneic GBM models (CT2A, GL261N4; C57BL/6 mice)	HSV-1 KOS strain (KG4:T124-GW backbone)	IL-12 transgene (CMV promoter); miR-124 targets in ICP4; gB NT mutations; gC-GFP	Intracranial (intratumoral)	None	↑ macrophage accumulation; ↑ CD8^+^, CD4^+^ T cells; enhanced TCR clonotype expansion; ↑ IFNG/IL18RA in T cells	Pro-inflammatory macrophage shift; ↓ microglia proportion; ↑ glioma-associated macrophages; myeloid gene changes (↑ CXCL9/10, SAA3)	CT2A: median 24.5 d, 17% LTS; GL261N4: median 33.5 d, 30% LTS; rechallenge protection	IL-12 oHSV reprograms TME and improves survival vs. unarmed oHSV
Grimes (2025) [20]	Syngeneic orthotopic GBM (GSC005, GL261-PVRL1; C57BL/6); analysis of human GBM trial datasets (G207, TCGA)	M002	Murine IL-12; γ134.5 deletion (R3659 control)	Intracranial (intratumoral)	None	Expansion of polyfunctional CD4^+^ T cells; ↑ IFNγ/TNFα/GzmB; ↓ PD-1^+^Lag-3^+^ exhausted CD4^+^; CD4-dependent control; IL6ra–Bcl6 axis	↑ MHCII on tumor/myeloid; ↓ Tregs/exhausted CD4^+^; “hotter” TME	GSC005: ~107 vs. 58 d (saline); benefit lost with CD4 depletion; rechallenge protection	IL-12 oHSV drives MHCII-dependent CD4 effector/memory responses enabling durable control
Saha (2018) [21]	Orthotopic GSC-derived GBM: human recurrent MGG123 (athymic) + murine 005 (C57BL/6)	G47Δ-mIL12	γ34.5 & α47 deletions; ICP6 inactivation; mIL-12 expression	Intratumoral (intracranial)	Axitinib; ±anti-CTLA-4	T-cell–dependent effects (immune-competent); ↑ CD3^+^/CD4^+^ with combo vs axitinib	↓ CD34^+^ vascularity; ↑ CD68^+^ macrophages; necrosis; ↓ Sox2^+^; ↓ PDGFR/ERK signaling	MGG123: median 42.5 d vs. 30–33; 005 benefit only immune-competent	Axitinib synergizes with IL-12 oHSV via anti-angiogenesis + immunity
Saha (2017) [22]	Immunocompetent orthotopic GBM (005 GSC, CT-2A; C57BL/6)	G47Δ-mIL12	γ34.5 & α47 deletions; ICP6 inactivation; mIL-12	Intratumoral (intracranial)	Anti-PD-1 + anti-CTLA-4	↑ CD8^+^ Teff; ↓ Tregs; ↑ Teff:Treg; CD4 & CD8 required; macrophage-dependent immunity	↑ macrophage infiltration + M1-like (↑ iNOS, pSTAT1); reduced immunosuppression	Triple therapy: ~89% LTS (005), 50% (CT-2A); rechallenge protection	IL-12 oHSV synergizes with dual ICB to eradicate GBM via macrophage + T-cell immunity
Bommareddy (2024) [23]	Orthotopic murine GBM: 005, CT-2A, GL261; athymic mice	G47Δ-mIL2	γ34.5 & α47 deletions; ICP6 inactivation; mIL-2 (mIL2-P2A-mCherry)	Intratumoral (intracranial)	±anti-PD-1; depletion/neutralization experiments	↑ CD8 infiltration; T-cell proliferation; efficacy dependent on CD4^+^; minimal systemic effects	Local IL-2; ↑ CD3^+^/CD8^+^; no ↑ Tregs/macrophages; no systemic IL-2	Survival ↑ in 005 (~63%) and CT-2A; no effect GL261; benefit lost with IL-2 neutralization/CD4 depletion	Intratumoral IL-2 via oHSV safely promotes T-cell immunity; CD4^+^ key determinant
Zhu (2022) [24]	U87MG/U87MG-Luc; PBMC-humanized NSG-B2m with orthotopic U87MG-Luc	oHSV-1 (HSV-1 F strain derived)	γ34.5 & ICP47 deletions	Intratumoral (intracranial); CAR T IV	CD70-CAR T	↑ IFN-γ; ↑ IL-6/IL-8/TNFα/TNFβ1; ↓ TGF-β1/IL-10/IL-4; ↑ CD4^+^IFN-γ^+^; ↓ Tregs	↑ CD4/CD8 TILs; ↑ NK; ↓ TGF-β1; pro-inflammatory shift	Tumor regression; ~42% complete disappearance; survival ↑ vs. mono	oHSV enhances CAR T efficacy by increasing infiltration and IFN-γ–driven TME reprogramming
Reale (2024) [25]	Syngeneic orthotopic GL261; C57BL/6	oHSV-1 (17syn+)	Δγ34.5; ΔUS12; EGFP inserted (UL55–UL56)	Intratumoral (intracranial)	None	↑ CD4^+^/CD8^+^ TILs; immune memory; ICD (↑ extracellular ATP); absence of PD-1^+^ TILs	↑ lymphoid/myeloid infiltration; ↑ MHC-II on myeloid; macrophage/microglia activation; astroglial reorg; vascular collapse/fibrosis	Median survival 38 vs. 26 d; 45% LTS; rechallenge protection	oHSV monotherapy converts cold → hot GBM, inducing durable systemic immunity
Jackson (2021) [26]	Syngeneic orthotopic CT2A, GL261N4; C57BL/6	KG4:T124; rQNestin34.5v1	KG4:T124: miR-124 ICP4 + gB NT mutations + gC-GFP; rQNestin34.5v1: nestin-driven ICP34.5, GFP-ICP6	Intratumoral (intracranial)	None	Immune recruitment in GL261N4: macrophages + CD4/CD8; ↑ PD-1^+^ T cells	Viral persistence ↔ macrophage/adaptive infiltration; CT2A returned to “cold” due to rapid clearance	Modest survival benefit in GL261N4; none in CT2A	Sustained intratumoral persistence required for immune recruitment/efficacy
Otani (2022) [27]	GL261N4, DB7, 005; C57BL/6, FVBN; recurrent GBM serum (*n* = 18)	rHSVQ; 34.5ENVE; rQnestin34.5	γ34.5-modified; nestin promoter control; luciferase reporter (rHSVQ-Luc)	Intratumoral (intracranial)	RO4929097 (GSI/NOTCH inhibitor)	oHSV activates NOTCH in myeloid; ↑ Jag1; ↑ CCL2/IL-10; MDSC recruitment; NOTCH blockade restores CD8 memory	NOTCH-driven immunosuppressive myeloid TME; GSI reverses recruitment + ↑ IFN-γ	Combo improves survival; ~44% complete responses GL261N4; rechallenge memory	oHSV-induced NOTCH limits efficacy; NOTCH inhibition reprograms TME and enhances durable immunity
Noh (2024) [28]	Orthotopic GBM/BCBM models (GBM12, 005, DB7, 4T1, MDA231Br); recurrent GBM patients (*n* = 14)	rHSVQ; oHSV-D11mt; rQNestin34.5v.2	oHSV-D11mt encodes secreted IGF2R domain 11 mutant Fc decoy; γ34.5/ICP6 deletions noted	Intratumoral (intracranial)	±anti-PD-L1; IGF2 neutralization; neutrophil depletion	↑ CD8 cytotoxicity; ↑ IFN-γ; T-cell memory; reversal of IGF2 suppression	↓ neutrophils/PMN-MDSCs; ↓ IL-10/TGF-β; ↑ pro-inflammatory milieu; blocks IGF2-IGF1R	Survival ↑ vs. rHSVQ/PBS; ~26% LTS	IGF2 mediates resistance; oHSV-mediated IGF2 blockade enhances antitumor immunity
Wirsching (2019) [29]	GEMM IDH-WT GBM (RCAS/tv-a XFM-Luc:PDGF, Cre; bilateral); C57BL/6	oHSVULBP3 (miR-124 attenuated)	miR-124 attenuation; human ULBP3; EGFP	Intratumoral (intracranial)	±anti-PD-1	↑ CD8 infiltration local + abscopal; ↑ central memory CD4/CD8; PD-1^+^ exhausted induced	TAM-dominant response; ↑ antigen presentation/TLR; ↑ MHC-II; abscopal TAM repolarization	Median survival 8 →18 d; contralateral tumor inhibition; more benefit + anti-PD-1	ULBP3-armed oHSV induces local/abscopal immunity and sensitizes to PD-1 blockade
Xu (2019) [30]	Human GBM xenografts (GBM30, U87ΔEGFR; nude); immunocompetent Gl261N4-hNectin1 (C57BL/6)	OV-CDH1 (parent OV-Q1)	ICP34.5 deletions; ICP6 inactivation; human E-cadherin (CDH1)	Intratumoral (intracranial)	None	Selective inhibition of KLRG1^+^ NK cytotoxicity; ↓ NK IFN-γ (KLRG1^+^)	↑ viral spread/load; ↑ NK/macrophage/microglia infiltration; enhanced fusion	Marked survival benefit; near-complete eradication (GBM30); benefit in immunocompetent model	E-cadherin oHSV evades NK clearance → better spread/efficacy without global NK suppression
Xu (2021) [31]	Xenografts (GBM43, GBM30; nude); immunocompetent CT2A-hCD47 (C57BL/6)	OV-αCD47-G1/-G4 (parent OV-Q1)	ΔICP34.5 × 2; ICP6 inactivation; full-length anti-CD47 IgG1 or IgG4 via T2A, IE4/5 promoter	Intratumoral (intracranial)	None	CD47–SIRPα blockade; IgG1 drives macrophage ADCP + NK ADCC; innate activation	↑ macrophage + NK infiltration; sustained local antibody; inflamed phagocytosis-permissive TME	OV-αCD47-G1 > OV-Q1 and > IgG4; many LTS (>125 d)	Locoregional anti-CD47 IgG1 via oHSV reprograms TME and improves efficacy with less systemic exposure
Monie (2021) [32]	LN229 inducible CCN1; CCLE GBM lines (*n* = 66); TCGA GBM (*n* = 174)	HSV-1 (context only)	None (in silico)	Not applicable	Not applicable	Identified CCN1-associated antiviral innate programs; STAT1/IRF7/DDX58 networks	CCN1-high state predicted immunoresistant/antiviral, reduced HSV permissiveness	Not applicable	CCN1-associated innate networks may mediate resistance to HSV-based virotherapy
Hong (2019) [33]	U87ΔEGFR; PDX neurospheres (GBM12, GBM30, GBM1016); intracranial xenografts (nude mice)	HSVQ		Intratumoral (intracranial)	HMGB1-blocking antibody (IP)	HMGB1 released as DAMP; blockade reduces inflammatory endothelial activation	↓ ICAM1; ↓ vascular permeability; ↓ RBC extravasation; ↓ edema (MRI T2)	Combo improves survival vs. oHSV alone; no change intrinsic growth rate	HMGB1 drives endothelial activation/edema after therapy; blocking HMGB1 improves outcome without impairing viral replication
Swanner (2023) [34]	Human lines (U251T3, LN229, U87ΔEGFR); PDX cells; intracranial xenografts (nude, NSG)	OVesRAGE (HSV-1 F strain; rHSVQ control)	γ34.5 deletion; ICP6 inactivation; esRAGE (IE4/5 promoter)	Intratumoral (intracranial)	None	Inhibits HMGB1–RAGE signaling; reduces EC activation	↓ ICAM1/VCAM1/CCL5; ↓ permeability/migration; ↑ viral replication/spread	Survival ↑ vs. rHSVQ; enhanced propagation	esRAGE oHSV mitigates HMGB1-RAGE EC activation, favors spread and improves efficacy
Passaro (2019) [35]	GL261N4, CT2A, CT2A/PD-L1; C57BL/6; xenografts U87ΔEGFR; nude mice	NG34scFvPD-1 (NG34 control)	γ34.5 & ICP6 deletions; human GADD34 under nestin promoter; CMV-driven secreted anti–PD-1 scFv	Intratumoral (intracranial)	None (vs. systemic anti–PD-1 comparator)	Local PD-1 blockade; T-cell–dependent immunity; immune memory	Limited replication; transient scFv; rejection immune-mediated	GL261N4 median 69 vs. 22 d; CT2A/PD-L1 ~17% LTS; rechallenge rejection	oHSV-delivered PD-1 scFv induces durable antitumor immunity despite limited replication
Tian (2022) [36]	GBM30-FFL xenografts (NSG/nude); CT2A-hEGFR (C57BL/6)	OV-Cmab-CCL5 (OV-Q1 parent)	ΔICP34.5; ICP6 inactivation; cetuximab–CCL5 fusion IgG1 (KIH Fc)	Intratumoral (intracranial); PBMC/activated T cells IV in humanized model	None	↑ NK/macrophage/CD4/CD8 migration & activation; ↑ NK ADCC, macrophage ADCP; ↑ IFN-γ, GzmB	Cold → hot conversion; ↑ innate/adaptive infiltration; abscopal effect; EGFR signaling inhibition	Survival ↑ vs. controls; benefit lost with T-cell depletion	Locoregional Cmab-CCL5 via oHSV potently reprograms TME and yields strong local/abscopal efficacy
Baugh (2024) [37]	U87; E57 GSCs; primary GBM aspirates; PBMC/T cell co-cultures	G207-NKG2D BiTE	G207 backbone; CMV-driven NKG2D–CD3 BiTE	In vitro	TMZ; radiation (2 Gy); TMZ + RT pretreatment	Antigen-independent T-cell activation; ↑ CD25/CD69/IFN-γ/GzmB/perforin; BiTE cytotoxicity	↑ NKG2D ligands after TMZ/RT; targets differentiated GBM + GSCs	Enhanced killing in vitro; potentiated by TMZ/RT	BiTE-armed G207 redirects T cells against GBM + resistant GSCs; synergizes with chemoradiation
Akl (2025) [38]	Human GBM (single-cell/spatial); murine orthotopic glioma (GL261, MK007; C57BL/6)	Engineered oncolytic HSV-1 vector	Local TRAIL blockade via scFv anti-TRAIL	Intratumoral (intracranial)	None	↓ T-cell apoptosis; ↑ tumor/virus-specific CD8; ↑ IFN-γ/TNF; ↓ CTLA-4	↓ TRAIL^+^STAT3^+^ astrocytes; ↑ CXCL9/10/11; TAM pro-inflammatory reprogramming	Tumor burden ↓; survival ↑ vs. control HSV	Astrocyte TRAIL suppresses immunity; oHSV-mediated TRAIL blockade reprograms microenvironment and improves outcomes
Ling (2023) [39]	Recurrent high-grade glioma/GBM (*n* = 41; 42 interventions)	CAN-3110 (rQNestin34.5v.2)	ICP34.5 under nestin promoter control	Intratumoral (stereotactic injection; iMRI-guided)	None	↑ intratumoral CD4/CD8; ↑ TCR diversity; public TCR clonotype dynamics; immune activation correlated with HSV-1 seropositivity	Immune activation signatures (T-cell/TH1); macrophage infiltration in perinecrotic regions; durable remodeling despite clearance in seropositive	Median OS 11.6 mo; HSV-1 seropositive OS 14.2 vs. 7.8 mo	Safe and immunogenic; benefit correlates with antiviral immune competence
Vazifehmand (2024) [40]	U251 glioblastoma cancer stem cells under hypoxia vs. normoxia	HSV-G47Δ	γ34.5 deletion; α47 deletion; ICP6 inactivation (G47Δ backbone)	In vitro infection (MOI = 1; 14 h)	None		Telomere/telomerase alterations: telomeres longer in normoxia after HSV-G47Δ, shorter in hypoxia; dysregulated hTERC/DKC1/TEP1; ↓ TERF2 both; MRN genes (↑ MRE11/RAD50 normoxia; RAD50 ↓ hypoxia)	Not reported	Telomerase/telomere complex may be targetable by HSV-G47Δ in both microenvironments

## Data Availability

No new data were created or analyzed in this study.
